# Robust Data Recovery in Wireless Sensor Network: A Learning-Based Matrix Completion Framework†

**DOI:** 10.3390/s21031016

**Published:** 2021-02-02

**Authors:** Manel Kortas, Oussama Habachi, Ammar Bouallegue, Vahid Meghdadi, Tahar Ezzedine, Jean-Pierre Cances

**Affiliations:** 1 The XLIM Research Institute, University of Limoges, 87000 Limoges, France; oussama.habachi@unilim.fr (O.H.); meghdadi@ensil.unilim.fr (V.M.); cances@ensil.unilim.fr (J.-P.C.); 2 SysCom Laboratory in the National Engineering School of Tunis, University of Tunis El Manar, Tunis 1002, Tunisia; ammar.bouallegue@enit.rnu.tn (A.B.); tahar.ezzedine@enit.rnu.tn (T.E.)

**Keywords:** Wireless Sensor Networks, Matrix Completion, data gathering, spatial data interpolation

## Abstract

In this paper, we are interested in the data gathering for Wireless Sensor Networks (WSNs). In this context, we assume that only some nodes are active in the network, and that these nodes are not transmitting all the time. On the other side, the inactive nodes are considered to be inexistent or idle for a long time period. Henceforth, the sink should be able to recover the entire data matrix whie using the few received measurements. To this end, we propose a novel technique that is based on the Matrix Completion (MC) methodology. Indeed, the considered compression pattern, which is composed of structured and random losses, cannot be solved by existing MC techniques. When the received reading matrix contains several missing rows, corresponding to the inactive nodes, MC techniques are unable to recover the missing data. Thus, we propose a clustering technique that takes the inter-nodes correlation into account, and we present a complementary minimization problem based-interpolation technique that guarantees the recovery of the inactive nodes’ readings. The proposed reconstruction pattern, combined with the sampling one, is evaluated under extensive simulations. The results confirm the validity of each building block and the efficiency of the whole structured approach, and prove that it outperforms the closest scheme.

## 1. Introduction

During the last decades, the Internet of Things (IoT) has emerged as a new business model that is composed of billions of communicating devices. Hence, it has gained considerable attention in both the scientific community and industry. However, the inclusion of the IoT into the fifth generation cellular systems (5G) and their evolution still represent a formidable technical challenge due to the huge number of sensors and the generated information. Note that one of the main challenges of the 5G is the massive connectivity for Machine-Type Communications (MTC) and managing its coexistence with the high rate continuous traffic that is generated by Human-Type Communications (HTC) in an efficient and effective manner. An interesting proposal is the Compressive Sensing (CS), which reduces the number of active agents at a given time slot, while remaining able to recover the sensing data. In general, Wireless Sensor Networks (WSNs) consist of a large set of sensor nodes, which are self-organising and geographically distributed across the network. They are usually used to monitor various physical phenomena with a high resolution, such as in forests, under water, as well as in civilian and habitat application areas. Usually, these devices operate in an unattended mode and they are unable to renew their batteries. Hence, energy efficiency is the main challenge for these networks, since it directly affects their lifetimes and, thus, their sustainability. In usual data gathering techniques, each sensor node takes measures and sends data to the sink node via multi-hop transmission. If nodes face packet losses, due to collisions or buffer overflows, packets are retransmitted, which leads to a high sensing cost and a heavy traffic, especially in large-scale networks. Indeed, reducing the number of transmitting source nodes, using techniques such as CS, is not only useful in reducing the collisions, but also crucial for sensor nodes that need to sleep to prolong their lifetimes.

Recently, it has been shown that the integration of the Matrix Completion (MC) technique, viewed as an extension of CS, has enhanced wireless networks scenarios. If the received data matrix has a low-rank structure, then it can be recovered with high accuracy while using the partially received elements [1]. Firstly, data are directly sensed in compressed form and the high energy-intensive recovery algorithm is executed at the sink node. Hence, the computation complexity is moved from sensor nodes to the sink. This meets well the resource-constrained devices and significantly reduces the energy consumption. Secondly, because MC handles the data in its matrix form, it can fully capture the signal correlation in both space and time dimensions and, hence, achieves a satisfying interpolation quality with a higher compression rate (very few transmitted readings).

In some applications, especially the densely deployed WSNs, the sensed data are, in general, highly correlated, and redundancy exists between the sensor nodes that belong to the same geographic area. These nodes can be arranged into a group or a cluster. Because they are monitoring the same targets or events, collecting raw data from all of the cluster members becomes inefficient and wasteful for the energy. Therefore, a sufficient subset of nodes can be selected from each group, according to a certain criteria, to be the representative of the whole network. These active nodes deliver their readings to the sink under a compression ratio that is guaranteed by MC theory, while the rest of nodes remain silent and do not participate in the sensing operation. Thus, as an extension to [2], we carry on with the twofold compression technique that has been updated compared to the paper [2]. First, we assume that part of nodes do not sense the environment at all. We can consider that these sensors are inactive or idle for a long period or that these nodes are absent. Specifically, in this paper, these notations are only related with the sensing activity, and all of the nodes are connected in order to participate in data forwarding (Here, a node is absent in the sense that its data reading is completely missing, and the sink node has to recover it correctly). The second compression level is that, at each time slot, only a subset of the active nodes, referred to as the transmitting ones, send their sensing data to the sink. Different from [2], the nodes having the higher correlation with other nodes, i.e., best represent the network, are selected as representative sensor nodes. Indeed, in order to be chosen as active nodes, they should be able to capture enough information regarding the others and the whole network. This strategy not only minimizes the energy cost and extend the network lifetime, but it also helps to avoid other problems, such as the traffic congestion collapse [3]. It is true that in [4], the choice of the active nodes follows a deterministic based metric. However, unlike [4], in this work, we explain, in detail, each building block of the introduced structured MC-based data gathering framework (the representative nodes selection process and the network clustering phase). Subsequently, we separately evaluate them in the numerical results section in order to illustrate the benefits of each building block of the proposed technique. Furthermore, in this paper, we propose a Multi-Gaussian signal model that introduces the solution of reproducing a signal retaining the behaviour of a given real world data by adjusting the correlation parameters. For that reason, this method represents an effective alternative to the real world signals.

The application of the just mentioned atypical high-loss scenario leads to a significant number of empty rows in the signal data matrix (a row (resp. column)) is called an empty row (resp. column) if and only if all of the values of the row (resp. column) are un-sampled), which completely disagrees with MC fundamentals. In fact, because MC approaches are based on the minimization of the matrix rank, they become useless when there is any empty row or empty column in the matrix. Indeed, MC techniques have been conceived to recover a matrix containing random missing elements [5]. In the state-of-art of MC-based algorithms, to the best of our knowledge, Ref. [6] is the only paper who dealt with the case, where there is a small number of missing rows in the received data matrix by applying a spatial pre-interpolation technique, which recovers data from neighboring sensor nodes. However, as the number of active nodes decreases, we also face absent nodes having absent neighbor sensors. Thus, this framework becomes unable to recover the data rows of these isolated sensor nodes. Hence, although this approach is interesting, it seems not well suited for the addressed scenario and it fails to take the existence of isolated sensor nodes (absent nodes having all their neighbors absent) into account. In this context, we develop our scheme, which, firstly, schedules the sampling pattern after efficiently identifying the different clusters and their representative nodes. Secondly, it treats the case of high compression ratios with a considerable number of inactive sensor nodes (empty rows) while using a sequence of three different interpolation techniques.

The proposed framework is also useful for another challenging scenario; when we have a small number of sensors that have to be deployed in a spacious area. Indeed, either the sensor nodes are costly or the environment is large enough to be content with the limited number of sensors. This may also concern the harsh environments that are difficult to access such as volcanoes and other troublesome environments, where the deployment of many sensor nodes is not practical and becomes expensive. However, in many applications, the amount of gathered data must be significant enough to be processed. The idea here is to place a relatively small number of spatially spaced sensor nodes to control the correlated field under a compression ratio. These sensor nodes represent other sensor nodes that do not really exist. Particularly, the sensory data field is, most of the time, highly correlated and redundant between nearby sensor nodes, which makes possible to estimate the readings at locations, where the signal cannot be sensed.

The main contributions of the paper are summarized, as follows:

- We generate a synthetic space-time signal that is composed of different Gaussians, each of which presents a cluster of wireless nodes. Like all the WSNs signals’ profiles, the generated signals are correlated in space and time, where spatial and temporal correlation parameters and models differ from one Gaussian to another and can be separately adjusted.
- For the sampling part, only a small subset of sensor nodes is selected to be active and report its readings. For each detected cluster, the active sensor nodes selection is achieved by considering the correlation criteria. Subsequently, for each time instant, we choose the transmitting sensor nodes with the same percentage from each cluster in order to ensure the diversity in the transmitted data, notably for the high compression ratios.
- For the reconstruction part, we propose using three different techniques to accurately rebuild the entire data matrix. In the first step, we fill the missing readings of the active sensor nodes by applying the MC. Subsequently, we carry on with the spatial pre-interpolation to handle a part of the empty rows while adjusting the 1-hop topology matrix to the presence of the disjoint clusters in the monitored field. Finally, we recover the rows of the isolated sensor nodes using a minimization problem interpolation-based technique with a spatial correlation matrix. In this paper, the third stage of data recovery pattern has been re-investigated and improved to be more efficient when compared to the one used in papers [2,4], i.e., providing a lower data recovery error for the isolated nodes. In the numerical results section, we evaluate the two techniques with respect to a tuning parameter, and we show that the proposed minimization problem interpolation-based method significantly enhances the data recovery performance.
- Through extensive simulations, we show that the proposed framework outperforms other existing techniques in the literature, especially when the number of inactive nodes increases.

The remainder of the paper is organized, as follows. The next section discusses the related work, and Section 3 provides a brief overview on the MC theory and introduces the problem formulation of the paper. Section 4 presents the signal model that we used for the evaluation of our approach. In Section 5, we introduce the efficient clustering method that we propose. In Section 6, we present a strategy that selects the set of the representative sensor nodes. Section 7 is dedicated to the data reconstruction framework. Before concluding the paper in Section 9, we carry out, in Section 8, extensive simulations in order to evaluate the performance of the proposed approach.

## 2. Related Work

Environmental WSN signal profiles exhibit both spatial and temporal dependency. Such structures generate redundancy and enable a succinct representation of the data while using a number of coefficients that are much smaller than its actual dimension. One popular postulate of such low-dimensional structures is sparsity, which is, a signal can be simply represented with a few non-zero coefficients in an invertible proper sparsifying domain [7]. CS has been introduced as a good fit for such application in both the acquisition and reconstruction of the signal [8]. With a number of measurements proportional to the sparsity level, CS enables the reliable reconstruction of the signal. Indeed, the latter can be encoded using a much lower sampling frequency than the traditional Nyquist one [9,10,11]. In order to handle the under-determined linear systems, efficient convex relaxation and greedy pursuit-based solvers have been proposed, such as NESTA [12], L1-MAGIC [13], and orthogonal matching pursuit (OMP) [14]. Over the past years, plenty of papers have addressed the data gathering problems in WSNs by the integration of the CS theory, which had made appealing progress in the network energy consumption [15,16,17,18,19,20,21].

Originally, CS-based schemes were designed to sample and recover sparse vectors, and they were classified either as purely spatial approaches [18,19,20,21,22] or as purely temporal ones [23]. Despite the incorporation of the kronecker CS framewok, the standard resolution of CS is still formulated in vector form [15,16,24,25,26]. Moreover, tools from linear algebra are still needed in order to reformulate the data matrix into the vector form. Without the need of computing an adaptive sparsifying basis, MC has recently emerged using another type of structural sparsity (a low-rank matrix holds singular values composing a sparse spectrum) [27], which is the matrix low rank property [1]. Because it treats the data matrix as a genuine matrix, MC can take advantage of the correlation in its two dimensions and capture more information. In [28], the authors have found that the data reconstruction performance of the MC depends on the compression ratio. In our previous work [29], we have illustrated that a simple MC-based approach requires a smaller fraction of sensor node readings. In [30], a state-of-the-art of MC-based algorithm for compressive data gathering has introduced the short-term stability with the low-rank feature. The considered feature was used not only to reduce the recovery error, but also to recover the likely empty columns appearing in the received data matrix. The existence of the empty columns was possible, since the readings were forwarded according to a presence probability. Differently, Zhou et al. in [31], have taken advantage of the temporal stability feature and a MC method based on the Bayesian inference to interpolate the missing data. Furthermore, the authors, in [32], addressed joint CS and MC. They have used the CS to compress the sensor node readings and then the MC to recover the non-sampled or lost information. However, this approach has not been compared to other state-of-the-art approaches to show its real contribution. In addition, they have not taken advantage of the space-time correlation of the signal as it should be, since they have used standard compression and sparsifying matrices for the CS. Different from [32], Wang et al. in [33], explored the graph based transform sparsity of the sensed data and considered it as a penalty term in the resolution of the MC problem. Similarly, Ref. [34] combined the sparsity and the low-rank feature in the decoding part, and, as in [33], has used the alternating direction method of multipliers to solve the constrained optimization problem. However, the authors have focused on vector-valued signals when sampling. In [35], the authors introduced an active sparse mobile crowd sensing approach that is based on MC with the intention to reduce the data acquisition cost, while inferring the non-sampled data readings. Because adaptability and efficiency are two very important issues in WSNs data gathering, Ref. [36] has proposed an adaptive and online data gathering scheme for weather data, purely based on MC requirements. In contrast to our proposed approach, this paper has addressed the sampling side differently. Indeed, they have focused on the sampled data locations in the received data matrix, whereas we have considered the sampled data locations in the network area.

The authors of [6] have focused on the case of MC recovery with the existence of successive data missing or corruption, which is referred to as structure faults. Indeed, they have considered that successive data may be missing or corrupted due to channel fading or sensor node failures, which creates successive missing data on rows and/or on columns. However, treating a significant number of totally missing rows was out of the scope of their paper. In this paper, we investigate how to solve a challenging problem in the WSNs: how to omit a considerable number of sensor nodes from the monitoring field and estimate their readings from the partially reported readings of the representative sensor nodes using a MC-based approach. It is worth mentioning that efficiently identifying the clusters, their representative nodes, as well as the data transmission schedule significantly affect the recovery accuracy.

## 3. Preliminary and Problem Formulation

### 3.1. Overview of Matrix Completion

As an extension of CS, the MC technique has emerged recently to benefit from the signal low-rank feature in order to recover the missing data from a substantially limited number of matrix entries [1]. That is, a partially unknown matrix $M\in {I\;R}^{N\times T}$ of rank r≪min{N,T} can be entirely reconstructed if a subset of its sampled elements $M_{ij}$ as well as their indices (i,j)∈Ω are available at the receiver side. The entry-wise partial observation operator $P_{\Omega }:{I\;R}^{N\times T}\to {I\;R}^{N\times T}$ is defined by the following expression:

(1) $${\left[P_{\Omega }\left(X\right)\right]}_{ij}=\left\{\begin{array}{c} X_{ij}\;\left(i,j\right)\in \Omega \\ \;0\;\mathrm{otherwise}. \end{array}\right.$$

Roughly speaking, the goal of the MC is to find a low-rank matrix *X* that is consistent with the observed measurements $M_{ij}$. According to [1], if Ω contains enough information and if $M\in {I\;R}^{N\times T}$ is a low rank matrix (to check whether the data matrix has a low-rank or approximately low-rank structure, one can perform the Singular Value Decomposition method [37]), we can fill the unknown entries by solving the following rank minimization problem:

(2) $$\mathrm{minimize}\;rank\left(X\right)\;s.t\;P_{\Omega }\left(X\right)=P_{\Omega }\left(M\right).$$

Yet, problem (2) is not convex, and algorithms solving it are doubly exponential. Fortunately, the nuclear norm ${\left\|X\right\|}_{*}$ minimization problem, which is a convex relaxation, can be solved. In fact, it is deployed as an alternative to the NP-hard rank minimization problem [38]. Thus, we have:

(3) $${\mathrm{minimize}\;\|X\|}_{*}=\sum_{i=1}^{r}\tau_{i}\left(X\right)\;s.t\;P_{\Omega }\left(X\right)=P_{\Omega }\left(M\right).$$

${\left\|X\right\|}_{*}$, which is also referred as the trace norm of *X*, denotes the sum of its singular values $\tau_{i}\geq 0$. In the literature, various efficient solvers for this type of systems have been suggested. For example, the Singular Value Thresholding (SVT) optimizes an approximation of (3) by adding a Frobenius-norm term to the objective function [39]:

(4) $${\mathrm{minimize}\;\tau \|X\|}_{*}+\;\frac{1}{2}{\left\|X\right\|}_{F}^{2}\;s.t\;P_{\Omega }\left(X\right)=P_{\Omega }\left(M\right).$$

Different from (3), another method has been proposed to approximate (2) rather than the nuclear norm, which is the matrix factorization. Low rank matrix fitting (LMaFit) [40], Sparsity Regularized SVD (SRSVD), and Sparsity Regularized Matrix Factorization (SRMF) [27] are among the approaches that use the matrix factorization method. These approaches are based on the fact that any matrix $X\in {I\;R}^{N\times T}$ of a rank up to *r* can be explicitly written as the product of two matrices with the form $X=LR^{tr}$, where $L\in {I\;R}^{N\times r}$ and $R\in {I\;R}^{T\times r}$. Hence, the goal here is to search over the set of rank-*r* matrices and find a point $LR^{tr}$ that is closest to the set of matrices, which meets *M* at all known entries. In order to solve the problem, an alternating minimization scheme is used by fixing one of *L* and *R* and making the other one as the optimization variable.

### 3.2. Problem Formulation

Consider a WSN that is composed of a set N={1,…,N} of *N* sensor nodes. Let $X\in {I\;R}^{N\times T}$ denote the data matrix that contains measurements that are collected by the set N during a sensing period of length *T* time slots. Precisely, the entry in the $i^{th}$ row and $t^{th}$ column of *X*, $x_{i,t}$ represents the $t^{th}$ data reading (t∈[1,T]) sensed by the $i^{th}$ node (i∈N). Both of the considered scenarios aim to estimate the full sensor nodes’ readings, *X*, through the use of a small subset $N_{rep}=\left\{1,\ldots ,N_{rep}\ll N\right\}$ of active sensors, being denoted by representative sensor nodes. It is worth mentioning that the number of active sensors is relatively small when compared to the number of inactive/inexistent ones. Specifically, decreasing the number of active sensors can likely generate a set of absent sensors that also have all their neighbors absent. We call them isolated (IS) sensor nodes.

We propose grouping together sensor nodes having similar readings in the same cluster while using a spectral clustering technique. In fact, the whole network is organized, as follows: $N={⋃}_{j=1}^{J}CL_{j}$ and $N=\sum_{j=1}^{J}cl_{j}$, where $cl_{j}$ is the number of sensor nodes that belong to $CL_{j}$ ($cl_{j}=card\left(CL_{j}\right)$), *J* is the number of detected clusters and $CL_{j}$ is the cluster *j*. Note that sensor nodes should capture enough information to be chosen as active. In the following, we define the node selection criterion, i.e., determine the nodes having the best presentation of the whole network. It will be shown in the sequel that the representative node selection as well as the data transmission schedule depend on the detected clusters.

To further reduce energy consumption, the representative sensors do not transmit their raw data to the sink. Instead, they trade on the data sensing along the *T* time instants and deliver a part of their readings according to a given compression ratio, which is, $m<N_{rep}$ readings rather than $N_{rep}$ readings per time slot. Consequently, the received data matrix $M\in {I\;R}^{N\times T}$ is composed of $N_{rep}$ partially empty data rows and $\left(N-N_{rep}\right)$ completely empty data rows. Note that, to replace any missing entry in *M*, we set a “zero” as a placeholder. We use a binary sample matrix $\Omega_{M}\in {I\;R}^{N\times T}$ that we call sensing and transmitting schedule to indicate, in each time slots *t*, which nodes sense and transmit its measurements. That is, $\Omega_{M(i,t)}=1$ if $x_{i,t}$ is available and 0 otherwise. Hence, the incomplete delivered data matrix *M* can be represented as the Hadamard product between $\Omega_{M}$ and *X*.

The first aim of our work is to well identify the matrix $\Omega_{M}$, as it represents the sampling schedule, which is of prime importance in the recovery performance.

The second aim of our work is to successfully recover all of the missing entries using a limited number of received readings. Therefore, we opted for the MC technique because of its numerous aforementioned benefits. Yet, the application of MC with the existence of a significant number of empty rows is still a challenging task to tackle, since the presence of empty rows or columns impedes the MC reconstruction. Thereby, in this paper we propose a novel interpolation technique that will be annexed to the MC one in order to recover the empty rows. It is noteworthy that the MC, as the first step in the reconstruction operation, is an important part since the performance of the subsequent proposed interpolation technique depends on the recovery accuracy of the MC.

**Figure 1:**
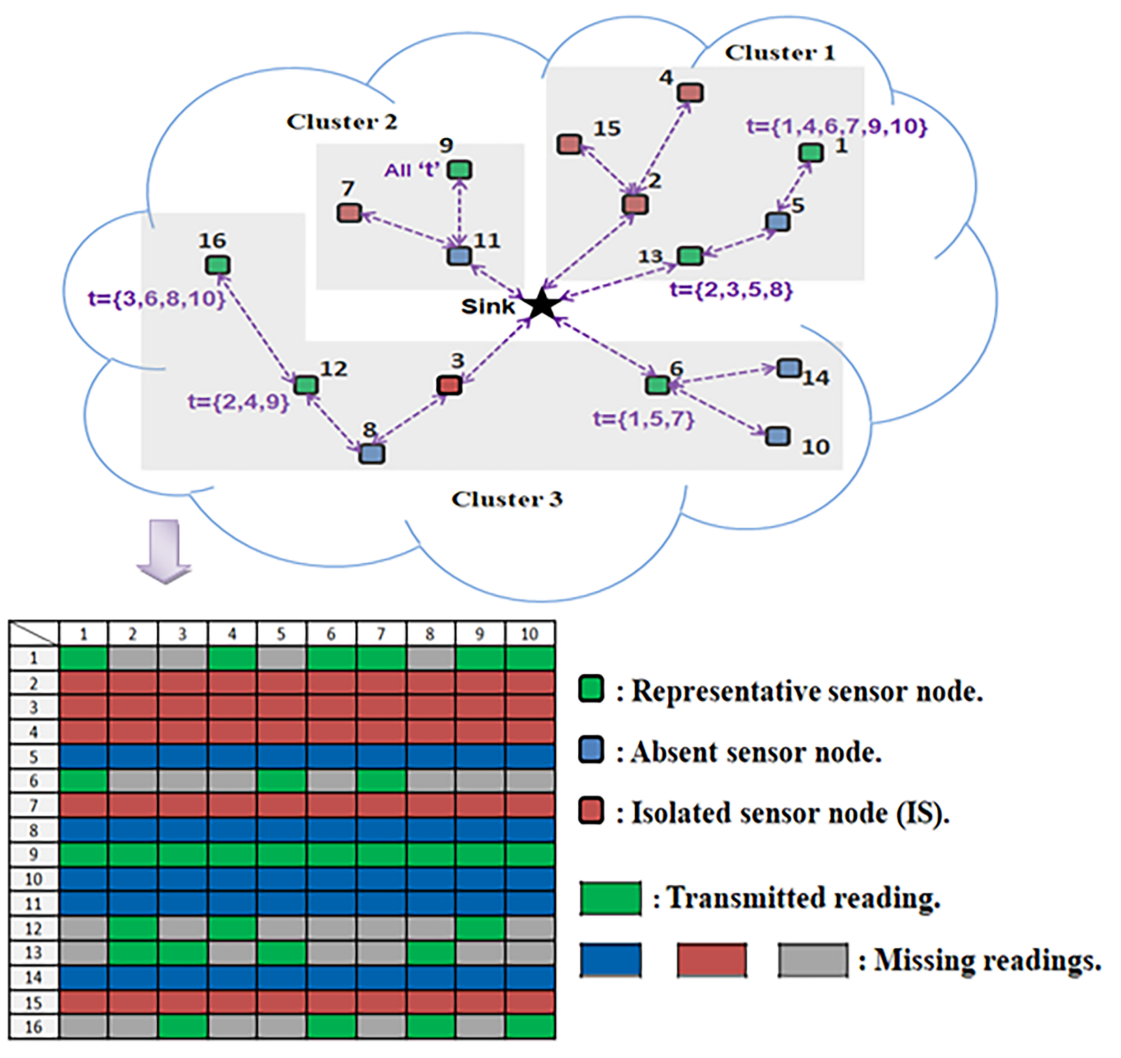
An illustrative miniature Wireless Sensor Network (WSN) with the resulting transmitted data matrix *M*.

Figure 1 illustrates an example of a WSN that consists of N=16 sensor nodes, among which $N_{rep}=6$ sensor nodes are selected to be active. The proposed combined reconstruction approach targets filling all of the missing entries corresponding to the non-transmitted readings.

## 4. Signal Model

In this section, we investigate the generation of a synthetic signal that is composed of different Gaussians, each of which presents a portion of the whole monitored geographic area. Because structure and redundancy in data are often synonymous with sparsity, which is analogous to low-rank [27], each portion of the signal is correlated in space and time, where the spatial correlation as well as the temporal correlation parameters differ from one Gaussian to another. These parameters can be separately adjusted because the corresponding functions are independent [41].

The proposed signal model is inspired from [41] that has introduced the solution of reproducing a signal retaining the behavior of a given real world data by adjusting the correlation parameters. In their model, all of the generated samples of the whole signal are Gaussian random variables with zero mean and unit variance. However, in this paper, we consider heterogeneous fields that are divided into a number of regions. Each one is modeled by a specific Gaussian (mean, variance) and different correlation characteristic. The number of different Gaussians as well as their distribution on the field can be fixed or defined according to the kind of signal that one wants to reproduce. This method represents an effective alternative to the real world signals.

In order to generate the signal of interest, we suppose that $D=\left[-x_{D},x_{D}\right]\times \left[-y_{D},y_{D}\right]$ is the space domain, where *x* and *y* are the space coordinates. Consider that we have *H* different regions, where $D_{h}$ is the space domain of region h=1,2,…,H, and $D={⋃}_{h=1}^{H}D_{h}$. Likewise, we suppose that the time is slotted into equal time slots t=1,2,….,T. Without a loss of generality, in Algorithm 1 we describe how to generate a correlated portion of the signal $z_{x}\left(p_{h},t\right):D_{h}\times T\to I\;R$ representing one region, where T is the time domain and $p_{h}$ is a point in (x,y) plane that corresponds to region *h*. The signal of the whole area is the combination of all the generated portions.

In order to obtain a spatially correlated signal, we apply to the signal, to be generated, a 2D filtering procedure using a specific correlation function rs(p), where p=(x,y). Among the numerous existing models in the literature, we generate the signal using the Gaussian filtering, as used in ([15] Equation (2)), which can be controlled by the parameter γ>0 (the Power Exponential model [41], when ν is equal to 2). The coloration of the signal with rs(p) has to be done in the frequency domain. Hence, before modeling the spatial correlation, a Fourier transformation is performed. Regarding the temporal correlation, the authors of [41] have used an autoregressive filter to enforce the temporal correlation in the signal model. Because the time is slotted into equal time slots, they only consider the one-step time correlation and use a simple coefficient ρ∈[0,1].
**Algorithm 1 **Model for generating a portion of the signal     **Input:** the generated field for t=1 : $w_{x}\left(p_{h},t\right)$, the temporal correlation parameter $\rho_{h}$, the spatial correlation parameter $\gamma_{h}$, the spatial correlation function computed in the frequency domain $Rs\left(\omega_{h}\right)=F\left(rs\left(p_{h}\right)\right)$.1:**for**t=1 to *T* **do**2:    **if** (t==1) **then**3:        $w_{y}\left(p_{h},t\right)=w_{x}\left(p_{h},t\right)-\eta_{h}$.4:    **else**5:        $w_{y}\left(p_{h},t\right)=\rho_{h}\times w_{y}\left(p_{h},t-1\right)+\sqrt{1-\rho_{h}^{2}}\times \varepsilon \left(p_{h},t\right)$, where $\varepsilon (p_{h},t)$ is a N(0,1) i.i.d random Gaussian noise.6:    **end if**7:    $W_{y}\left(\omega_{h},t\right)=F\left(w_{y}\left(p_{h},t\right)\right)$.8:    $Z_{y}\left(\omega_{h},t\right)=W_{y}\left(\omega_{h},t\right)\times Rs{\left(\omega_{h}\right)}^{1/2}$.9:    $z_{y}\left(p_{h},t\right)=F^{-1}\left(Z_{y}\left(\omega_{h},t\right)\right)$.10:  $z_{x}\left(p_{h},t\right)=z_{y}\left(p_{h},t\right)+\eta_{h}$.11:**end for**     **Output:** the space–time correlated signal portion $z_{x}\left(p_{h},t\right)$ of zone $D_{h}$.

To start the signal generation process, for t=1, we define $w_{x}\left(p_{h},t\right):D_{h}\times T\to I\;R$ to be an i.i.d random Gaussian. That is, for any specific position $p_{h}\left(x,y\right)$, $w_{x}\left(p_{h},t\right)$ is a Gaussian random variable with mean $\eta_{h}\in I\;R$ and unit variance. Algorithm 1 describes how to produce a portion of the whole signal $z_{x}\left(p,t\right):D\times T\to I\;R$, which represents the (x,y) signal. By construction, $z_{x}\left(p,t\right)$ is a three-dimensional (3D) matrix of size $\left(2y_{D}\times 2x_{D}\times T\right)$. The data matrix of interest, *X*, denotes the two-dimensional (2D) signal that is discretized by the *N* sensor nodes along the *T* time slots.

**Figure 2:**
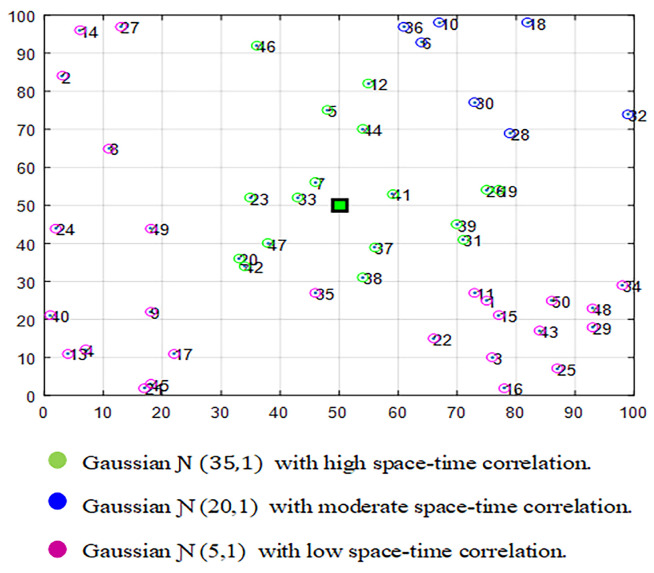
An example of a monitored area composed of three portions, each of which is presented by a different Gaussian.

Figure 2 illustrates an example of an area of size 100 m×100 m that is monitored by N=50 sensor nodes. We can notice, through the colors, that this field is divided into three different regions (H=3) that are presented by three different Gaussians.

## 5. Clusters Detection

In this section, we investigate the partition of the deployed sensor nodes into *J* clusters. The main reason for partitioning the nodes is to involve all of the detected clusters in the data sensing and transmission. It is well-known, in the conventional MC, that transmitting sensors are selected in a random way during the *T* time slots. This kind of selection can disregard sensors that belong to the small clusters, which deteriorates the recovery process. However, if we make all of the clusters contribute in the data transmission process, then we fortify the diversity in the delivered data set. Therefore, for each *t*, according to a given compression ratio and using the same percentage, a set of sensor nodes is picked from each cluster to form the sampling and transmission schedule. It will be shown, in the simulation section, that taking the detected clusters during the sampling process into account significantly enhances the data recovery performance, especially for the high compression ratios. Indeed, our aim is to partition the sensor nodes into different clusters in such a way that we attempt to maximize the intra-cluster similarities and minimize the inter-cluster similarities. Such a successful grouping can be achieved while using the Normalized Spectral Clustering

Usually, sensor nodes, which are situated spatially close to each other, have similar readings. Nevertheless, there are some cases, where nearby nodes are separated by a certain barrier and they have readings relatively different from each other. Given the example of sensor nodes deployed in a city to monitor the air pollution. Suppose that we have a public garden located next to a road. Hence, the nearby nodes, which are placed on different sides of the borders, do not necessarily have similar readings. Therefore, to cluster the nodes, the sink relies on their delivered readings (at the initialization, we let all of the sensor nodes send their information during a short learning period $T_{lp}\ll T$) and considers the set of data vectors, $\chi_{lp}=\left\{x_{lp\;1}^{tr},x_{lp\;2}^{tr},\ldots ,x_{lp\;N}^{tr}\right\}$, which we want to partition into *J* clusters. $x_{lp\;i}\in {I\;R}^{1\times T_{lp}}$, viewed as a $T_{lp}$-dimensional data points, holds the readings that are sent by the sensor node *i* during the learning period. The spectral clustering technique performs data clustering and treats it as a graph partitioning problem without setting any assumption on the clusters form. It transforms the given set $\chi_{lp}$ into a weighted graph G=(V,E) while using some notion of symmetric similarity matrix $A\in {I\;R}^{N\times N}$, where each vertex $v_{i}$ represents $x_{lp\;i}$, and each edge between two vertices $v_{j}$ and $v_{i}$ represents the similarity $a_{j,i}\geq 0$. It is recommended to use the Normalized Spectral Clustering, as mentioned above. Hence, we implemented the NJW algorithm [42] (the algorithm name, NJW, is attributed according to the authors’ names, which is, Ng, Jordan, and Weiss), which is detailed in Algorithm 2.

Commonly, identifying the number of clusters *J* in an optimal manner is the main concern of all clustering algorithms. Generally, with spectral clustering, we find the number *J* by analyzing the Laplacian matrix eigenvalues that are computed using *A* and according to the chosen clustering method. In this work, we choose to apply the eigengap heuristic [43], which defines *J* by finding a drop in the magnitude of Laplacian eigenvalues, $\left\{\lambda_{1},\lambda_{2},\ldots ,\lambda_{N}\right\}$, sorted in increasing order. That is:

(5) $$J=arg\;\;\max_{i}\left(\lambda_{i+1}-\lambda_{i}\right).$$

The idea here is to pick the number *J* in such a way that all of the Laplacian eigenvalues $\lambda_{1},\ldots ,\lambda_{J}$ are very small when compared to $\lambda_{J+1}$, which marks relatively a large value.

Regarding the similarity matrix *A*, we opted for the Gaussian kernel to measure the similarity between the data points $\left\{x_{lp\;i}\right\}$ [42], where σ is a scaling parameter that controls the neighborhoods width:

(6) $$a_{i,j}=\exp\left(-\frac{∥x_{lp\;i}-x_{lp\;j}{∥}^{2}}{2\sigma^{2}}\right).$$

According to ([42] Theorem 2), an appropriate σ can be automatically fixed after repeatedly running the algorithm while using a number of values and choosing the one that forms the least distorted partition in the spectral representation space. In order to determine the appropriate parameter σ, in ([43] Section 8), the authors had provided several rules of thumb that are frequently used. As an example, the method that we have used states that σ can be chosen to be in the order of nearly the mean distance of a point to its $k_{m}^{th}$ nearest neighbor, where $k_{m}∼log\left(N\right)+1$.
**Algorithm 2 **The Ng, Jordan, and Weiss (NJW) Spectral Clustering algorithm     **Input:** The set of data vectors $\chi_{lp}=\left\{x_{lp\;1}^{tr},x_{lp\;2}^{tr},\ldots ,x_{lp\;N}^{tr}\right\}$, the number *J* of clusters to detect according to (5).Pre-processing:1:Calculate the similarity matrix *A* according to (6).2:Calculate the degree matrix *D*, which is a diagonal matrix defined by : $d_{i,i}=\sum_{j=1}^{N}a_{i,j}$.Spectral representation:3:Compute the normalized graph Laplacian matrix $L_{sym}=D^{-1/2}\left(D-A\right)D^{-1/2}$.4:Proceed the eigenvalues decomposition of $L_{sym}$ and find the *J* eigenvectors corresponding to the smallest eigenvalues, arranged in increasing order.5:Form the matrix *U* by stacking the *J* eigenvectors in columns: $U=\left[u_{1},\ldots ,u_{J}\right]\in {I\;R}^{N\times J}$.6:Normalize the *U*’s rows to norm 1 in order to get the matrix $U_{n}\in {I\;R}^{N\times J}$ , that is, $U_{n_{i,j}}=u_{i,j}/{\left(\sum_{j}u_{i,j}^{2}\right)}^{1/2}$.Clustering:7:Treat each row of $U_{n}$, ${\left(u_{n_{i}}\right)}_{i=1,\ldots ,N}$, as a data point in ${I\;R}^{J}$, then partition them into *J* subgroups, $Q_{1},\ldots ,Q_{J}$, using *k*-means algorithm.8:Attribute the original points $x_{lp\;i}$ to cluster *j* if and only if row *i* of the matrix $U_{n}$ was attributed to cluster *j*.     **Output:** Clusters $CL_{1},\ldots ,CL_{J}$ with $CL_{j}=\left\{i|u_{n_{i}}\in Q_{j}\right\}$.

**Figure 3:**
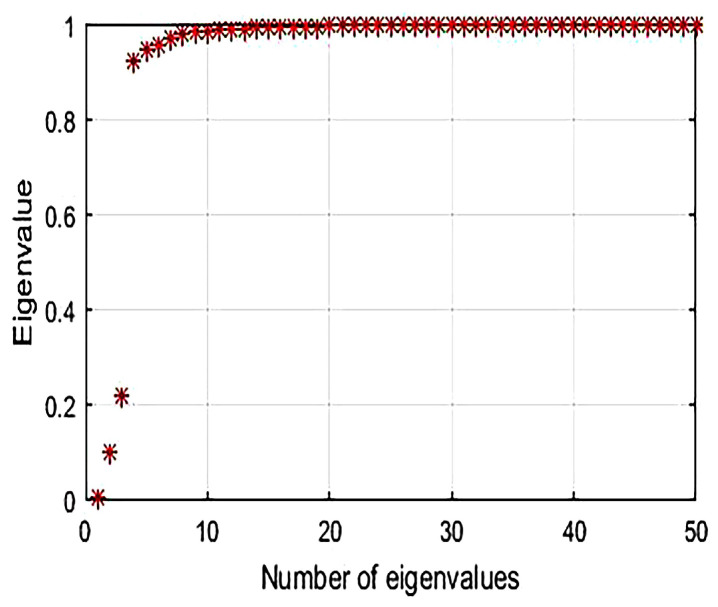
The Laplacian matrix eigenvalues of a signal that is sensed from the monitored area of Figure 2.

Figure 3 plots the sorted eigenvalues of the normalized Laplacian matrix that is computed from the generated signal of the example of Section 4 while using the first four steps of the aforementioned clustering algorithm. Clearly, there is a relatively large gap between the 3rd and 4th eigenvalue of this trace. According to metric (5), the data set contains three clusters, which is well approved.

## 6. Sampling Pattern

In this section, we determine how the correlation criteria can be considered to select the representative sensor nodes and how we take the detected clusters in the selection process as well as in the sensing and transmission schedule into account. Unlike our previous work [2], where the set $N_{rep}$ of representative sensor nodes is randomly chosen, in this paper $N_{rep}$ must hold enough information towards the other nodes to be chosen as representative of the network. Relying on the Enhanced Correlation Based Deterministic Node Selection (ECB-DNS) procedure, which was used in previous works [15,16], the active sensor nodes selection is achieved by considering the inter-spatial correlation, which is computed through the conditional variances of the sensor nodes. This technique enables selecting the sensor node $g^{*}$ holding the maximum informative value $m^{\prime }$ with respect to the set $S_{1}$ of sensor nodes that are not selected yet. Namely:

(7) $$g^{*}=arg\;\max_{g\in S_{1}}\left(m_{g}^{\prime }\right),\;\mathrm{where}\;m_{g}^{\prime }=\sum_{i\in S_{1}}\frac{\sigma_{ig}^{2}}{\sigma_{g}^{2}}.$$

In (7), $\sigma_{ig}$ represents the covariance between the reading $x_{i}$ of sensor *i* and the reading $x_{g}$ of sensor *g*, whereas $\sigma_{g}^{2}$ presents the variance of $x_{g}$. It is noteworthy that the way of exploiting this technique in our approach is different to that in [15]. According to their scenario, all of the *N* nodes contribute to the data sensing and transmission over the *T* time slots, while, in this approach, only $N_{rep}\ll N$ nodes are selected to be active and represent the *J* detected clusters. In order to cover all of the clusters, the set $N_{rep}$ consists of the combination of *J* subsets, ${\left(N_{rep_{j}}\right)}_{j=1,\ldots ,J}$, where $N_{rep_{j}}$ includes $N_{rep_{j}}$ representative nodes picked from cluster $CL_{j}$ while using the same shared percentage $pct_{Nrep}$. That is:

(8) $$N_{rep}=\sum_{j=1}^{J}N_{rep_{j}},\;\mathrm{where}\;N_{rep_{j}}=pct_{Nrep}\%\times cl_{j}.$$

In (8), if $pct_{Nrep}\%\times cl_{j}$ is not an integer, we round $N_{rep_{j}}$ to the nearest integer greater than or equal to the value of that element. Here, the selection of the sets $N_{rep_{j}}$ of clusters’ representative nodes is independent from one cluster to another. Hence, the set $S_{1}$ that appears in expression (7) is replaced by the set $S_{1}^{j}$, which represents the sensor nodes of the cluster $CL_{j}$ that are not yet selected. Thus, we have:

(9) $$g^{*}=arg\;\max_{g\in S_{1}^{j}}\left(m_{g}^{\prime }\right),\;\mathrm{where}\;m_{g}^{\prime }=\sum_{i\in S_{1}^{j}}\frac{\sigma_{ig}^{2}}{\sigma_{g}^{2}}.$$

The selection process is the same for the *J* sets $N_{rep_{j}}$. Thus, for each cluster $CL_{j}$, according to (9), at each iteration $n\in \{1,\ldots ,N_{rep_{j}}\}$, a sensor node $g^{*}\left(n\right)$ is selected and moved from set $S_{1}^{j}$ to set $S_{2}^{j}$. Note that $S_{2}^{j}$ represents the set of nodes of cluster $CL_{j}$ that are already chosen during the previous iterations. Once a sensor $g^{*}\left(n\right)$ is put in $S_{2}^{j}$, the metric $m^{\prime }$ of the remaining sensors of set $S_{1}^{j}$ should be recomputed in order to prepare the selection of the next sensor node $g^{*}\left(n+1\right)$. Here, by removing $g^{*}\left(n\right)$ from $S_{1}^{j}$, we cancel its impact on the rest of the nodes in $S_{1}^{j}$. Hence, the selection of the sensor node $g^{*}\left(n+1\right)$ will be achieved as if the sensor node $g^{*}\left(n\right)$ did not exist in the network. The node selection process, especially the manner in how we remove the correlation effect of node $g^{*}\left(n\right)$ from $S_{1}^{j}$, follows the steps that are outlined in Algorithm 3. For the initialization, we define the data matrix sent during the learning period $X_{lp}={\left[x_{lp\;1}^{tr},x_{lp\;2}^{tr},\ldots ,x_{lp\;N}^{tr}\right]}^{tr}\in {I\;R}^{N\times T_{lp}}$ that we partition into *J* sub-matrices $X_{lp}^{j}\in {I\;R}^{cl_{j}\times T_{lp}}$, where $X_{lp}^{j}$ holds data sent by nodes belonging to $CL_{j}$. Besides, we assume that the spatial correlation feature inherent in $X_{lp}$ reflects that in *X*. By analogy with [15], the computational complexity of selecting $N_{rep_{j}}$ representative nodes from $CL_{j}$ is $O(N_{rep_{j}}cl_{j})$. However, different from [15], where in each time slot *t*, a new and a different set of active transmitting source nodes should be found using the node selection metric, in this work, the selection of the representative nodes’ set is performed only once, at the beginning of the sensing period *T*.

Given the example of Figure 1, we can note the existence of three detected clusters within the network. We suppose that $pct_{Nrep}=30$. Thus, 30% of nodes will be selected from each cluster to be active. That is to say that we should pick $N_{rep_{1}}=2$ sensors from $CL_{1}$, $N_{rep_{2}}=1$ sensor from $CL_{2}$ and $N_{rep_{3}}=3$ sensors from $CL_{3}$. That is, in total $N_{rep}=6$ representative sensors. Based on the correlation among the sensor nodes and using Algorithm 3, the obtained subsets are as follows: $N_{rep_{1}}=\left\{13,1\right\}$, $N_{rep_{2}}=\left\{9\right\}$ and $N_{rep_{3}}=\left\{12,6,16\right\}$.

Once the set $N_{rep}$ of representative sensor nodes is defined, the sink focuses on the sensing and transmitting schedule, $\Omega_{M}$, by assigning *m* transmitting nodes for each time instant *t*. Obviously, these nodes are picked from the set $N_{rep}$. As has been stated in the previous section, in order to ensure the diversity in the delivered data, the *m* transmitting nodes are chosen in such a way that we randomly pick, with the same shared percentage $pct_{m}$, $m_{j}$ nodes from each subset $N_{rep_{j}}$ corresponding to cluster $CL_{j}$. Likewise (8), we have:

(10) $$m=\sum_{j=1}^{J}m_{j},\;\mathrm{where}\;m_{j}=pct_{m}\%\times N_{rep_{j}}.$$

Let us focus again on the example of Figure 1, we suppose that $pct_{m}=20$. Thus, for each *t*, 20% of sensors from each subset $N_{rep_{j}}$ are randomly designated to deliver their data to the sink. Because the used number *N* of this example is very small, we end with $m_{j}=1$ transmitting sensor from each cluster for each *t*.

To conclude, rather than selecting, in a purely random way, the measurement locations, as usually used in the conventional MC method, in this section we presented how to intelligently assign transmitting sensor nodes that can well represent the network relying on their correlations.
**Algorithm 3 **A cluster representative sensor nodes selection process     **Input:** For j=1,…,J, $S_{1}^{j}=CL_{j}$, $S_{2}^{j}=\left\{\emptyset \right\}$, $N_{rep_{j}}=\left\{\emptyset \right\}$, $X_{1}^{j}=X_{lp}^{j}$, a zero-vector $X_{2}^{j}\in {I\;R}^{1\times T_{lp}}$, n=1.1:**for**n=1 to $N_{rep_{j}}$ **do**2:    **if** (n==1) **then**3:        Compute the covariance matrix $\Sigma^{j}\in {I\;R}^{cl_{j}\times cl_{j}}$ of $X_{lp}^{j}$.4:        According to (9) and using $\Sigma^{j}$, compute the metrics $m^{\prime }$ then select $g^{*}\left(n\right)$.5:        Remove the reading $x_{lp\;g^{*}\left(n\right)}^{j}$ of node $g^{*}\left(n\right)$ from $X_{1}^{j}$ so that it becomes $X_{1}^{j}=\left[x_{lp\;1}^{j},x_{lp\;2}^{j},\ldots ,x_{lp\;g^{*}\left(n\right)-1}^{j},x_{lp\;g^{*}\left(n\right)+1}^{j},\ldots ,x_{lp\;cl_{j}}^{j}\right]\in {I\;R}^{cl_{j}-n\times T_{lp}}$ and $X_{2}^{j}$ takes the values of node $g^{*}\left(n\right)$ so that $X_{2}^{j}=x_{lp\;g^{*}\left(n\right)}^{j}$.6:        Following that removal, $\Sigma^{j}$ can be written as:
$$\Sigma^{j}=\left[\begin{array}{cc} \Sigma_{1,1}^{j} & \Sigma_{1,2}^{j}\\ \Sigma_{2,1}^{j} & \Sigma_{2,2}^{j} \end{array}\right],$$
where $\Sigma_{1,1}^{j}\in {I\;R}^{cl_{j}-n\times cl_{j}-n}$ is the covariance matrix of $X_{1}^{j}$, $\Sigma_{1,2}^{j}=\Sigma_{2,1}^{j^{tr}}\in {I\;R}^{cl_{j}-n\times 1}$ is the covariance vector between $X_{2}^{j}$ and $X_{1}^{j}$, and $\Sigma_{2,2}^{j}$ is the variance of $X_{2}^{j}$.7:    **else if** (n≥2) **then**8:        Following the removal of node $g^{*}\left(n-1\right)$ from $S_{1}^{j}$, re-compute the conditional covariance matrix of $X_{1}^{j}$ knowing $X_{2}^{j}=x_{lp\;g^{*}\left(n-1\right)}^{j}$; $\Sigma_{1,1|2}^{j}\in {I\;R}^{cl_{j}-\left(n-1\right)\times cl_{j}-\left(n-1\right)}$ where:
$$\begin{array}{cc} \Sigma_{1,1|2}^{j} & =\Sigma_{1,1}^{j}-\Sigma_{1,2}^{j}{\left(\Sigma_{2,2}^{j}\right)}^{-1}\Sigma_{2,1}^{j}. \end{array}$$9:        According to (9) and using $\Sigma_{1,1|2}^{j}$, re-compute the metrics $m^{\prime }$ then select $g^{*}\left(n\right)$.10:      $\Sigma^{j}$ takes the values of $\Sigma_{1,1|2}^{j}$.11:      Perform step 5 then step 6.12:    **end if**13:    $S_{1}^{j}=CL_{j}\backslash \left\{g^{*}\left(n\right)\right\}$ and $g^{*}\left(n\right)\in S_{2}^{j}$.14:**end for**     **Output:** $N_{rep_{j}}=S_{2}^{j}$.

## 7. Reconstruction Pattern

After revealing in detail how to select the $N_{rep}$ representative sensor nodes and how to schedule their participation in the data sensing and transmission, in this section we focus on how to approximate the entire N×T data matrix *X* based on the limited amount of reported readings. Isolating $\left(N-N_{rep}\right)$ inactive sensor nodes from the sampling and transmission schedule entails the existence of $\left(N-N_{rep}\right)$ fully empty rows in the received data matrix $M\in {I\;R}^{N\times T}$, which impedes the MC technique that is completely unable to estimate the original matrix. Therefore, the use of other complementary interpolation techniques becomes needed. In this context, we develop a structured MC-based recovery algorithm that is able to ensure the reconstruction of the entire N×T data matrix *X*.

**Stage 1:** obviously, it is not feasible to directly apply the MC technique with the existence of $\left(N-N_{rep}\right)$ fully empty rows. Therefore, we have to remove these rows from *M*. We denote the resultant matrix as $M_{MC}\in {I\;R}^{N_{rep}\times T}$, containing the partially delivered readings of the representative sensor nodes. We carry on with the same removal from $\Omega_{M}$ to obtain $\Omega_{MC}\in {I\;R}^{N_{rep}\times T}$. Subsequently, making use of the solution introduced in (4) or any other method proposed for the MC resolution, we fill the missing entries of $M_{MC}$ that correspond to the non-transmitted data readings of the $N_{rep}$ sensor nodes. The threshold parameter τ roughly equals 100 times the largest singular value of $M_{MC}$, as has been introduced in [39]. We denote $X^{\prime }\in {I\;R}^{N_{rep}\times T}$ as the combination of the MC based estimation and directly observed data. Finally, we update $X^{\prime }\in {I\;R}^{N\times T}$ by adding the $\left(N-N_{rep}\right)$ empty rows and then placing them in their proper corresponding locations of *M*.

**Stage 2:** after filling the random missing readings, leave the $\left(N-N_{rep}\right)$ completely missing rows that correspond to the inactive sensor nodes. In this phase, we carried on with the spatial pre-interpolation technique of [6], which rebuilds the data of an empty row relying on the available data of the neighboring sensor nodes. To apply this method, they used a kind of an N×N binary symmetric matrix *Y* that they called a 1-hop topology matrix, where both of the columns and rows denote the sensor nodes. The sink assigns 1 to Y(i,j) and Y(j,i) if it finds that sensor node *i* and sensor node *j* are 1-hop neighbors. However, according to the signals nature that we consider, and to avoid untrustworthy data reconstruction, we consider that, even though two sensor nodes are geographically close to each other, if they do not belong to the same cluster, then they are not considered to be neighbors.

The number $N_{rep}$ of the active sensor nodes is very small when compared the total number *N*, which means that the $\left(N-N_{rep}\right)$ inactive sensor nodes constitute the preponderant portion of the network, as mentioned before. Consequently, there are several IS nodes in the network (having all of their neighbors absent). Hence, with the use of the stated topology matrix *Y*, this interpolation technique can achieve the data reconstruction only for the absent sensor nodes that have neighbors that belong to $N_{rep}$. We suppose that the network distribution contains $N_{Is}$isolated sensor nodes. Subsequently, the resulting data matrix $X^{\prime \prime }\in {I\;R}^{N\times T}$, obtained at the end of this stage, still holds $N_{Is}$ empty rows to be recovered ($N_{Is}$ all-zeros rows). For the detailed steps of the above interpolation method, the reader may refer to ([6] Section VI) (As for the complexity of the used spatial pre-interpolation, according to [6], it is estimated to be very low, since this technique is based on simple matrix multiplication with neighbor information).

**Stage 3:** since the above interpolation technique is limited to recover only a part of the total empty rows (absent nodes), we resort to a second spatial interpolation to rebuild the remaining part of the empty rows (isolated nodes). Benefiting once again from the spatial dependency among the sensor nodes, we fill the remaining empty rows while using the following minimization problem:

(11) $$\mathrm{minimize}\;(fac_{1}\times ∥\hat{X}\left(\bar{\Omega_{IS}},:\right)-X^{\prime \prime }\left(\bar{\Omega_{IS}},:\right){∥}_{F}^{2}+fac_{2}\times ∥S\times \hat{X}{∥}_{F}^{2}),$$

where $\bar{\Omega_{IS}}$ denotes the set of indexes of the non isolated nodes, i.e., the representative nodes and the absent ones. *S* represents the spatial constraint matrix, whose computation steps will be detailed hereafter, $fac_{1}$ and $fac_{2}$ are two tuning parameters, and $\hat{X}\in {I\;R}^{N\times T}$ is the final reconstructed data matrix. It is noteworthy that the above proposed minimization-based interpolation technique has been updated when compared to the one of our previous works ([2] Equation (8)) and ([4] Equation (5)), and, through simulations, we found out that the updated minimization significantly enhances the data reconstruction quality of the isolated nodes. Note that the resolution of this optimization problem can be easily accomplished while using the semidefinite programming (SDP). We opted for the CVX package [44], implemented in Matlab, as an advanced convex programming solver, in order to solve (11) and obtain $\hat{X}$.

In this equation, the matrix $S\in {I\;R}^{N\times N}$ relatively reflects our knowledge regarding the spatial structure inherent in the data, since it is computed based on the learning data matrix $X_{lp}\in {I\;R}^{N\times T_{lp}}$. This spatial matrix expresses the similarities between the sensor nodes’ readings. Suitably, we use the Euclidean distance as a distance function in the data domain of the sensor nodes to model the similarity between the rows of $X_{lp}$, whereby the smaller the distance between two rows, the closer they are. Below are the steps to obtain *S*:

1—We initiate these steps with an all-zeros matrix *S*.

2—The similarity between the rows in $X_{lp}$ is not evident as the ordering of the sensor nodes’ indexes in $X_{lp}$ is arbitrary. Thus, for each row *i* of $X_{lp}$, we search for the set $j_{i}^{\prime }$ of indexes of the *K* closest rows to *i*, which is, $j_{i}^{\prime }=\left\{j_{k}\neq i\;|\;k=1,...,K\right\}$.

3—Assuming that the row *i* can be approximated through the linear combination of the rows of set $j_{i}^{\prime }$, we perform the linear regression to compute the weight vector $W=\left[w\left(1\right),\ldots ,w\left(K\right)\right]\in {I\;R}^{1\times K}$ through the following equation:

(12) $$W=X_{lp}\left(i,:\right)\times X_{lp}{\left(j_{i}^{\prime },:\right)}^{tr}\times {\left[X_{lp}\left(j_{i}^{\prime },:\right)\times X_{lp}{\left(j_{i}^{\prime },:\right)}^{tr}\right]}^{-1}.$$

4—Finally, we assign 1 to S(i,i) and −w(k) to $S(i,j_{k})$.

As soon as these steps have been carried out for all the rows *i*, we obtain the matrix *S*, with which we interpolate $\hat{X}$, as in (11) (here, since, for each row of $X_{lp}$, we search for the set $j_{i}^{\prime }$, while using a simple Euclidean distance, the complexity is O(N). Moreover, performing the linear regression in (12) to compute the weight vector *W* is basically dominated by simple multiplication and division operations of matrix, which makes the complexity low).

Now, there remains the last adjustment to realize, that is, the scaling of the two parameters, $fac_{1}$ and $fac_{2}$ of (11). The regularization parameters $fac_{1}$ and $fac_{2}$ are introduced in order to establish a trade-off between a close fit to the matrix $X^{\prime \prime }$ and the intention of fulfilling the $N_{Is}$ remaining empty rows while using *S*. Through several simulations, we found that adjusting these parameters nicely improves the reconstruction performance, and the found values of $fac_{1}$ and $fac_{2}$ are independent of the size of the matrix (*N* and *T*) as well as the Gaussians’ values composing the synthetic signal.

Let us focus again on the example shown in Figure 1. The dotted lines refer to the neighborhood relation between sensors. As we can see, the sensors {5,8,10,11,14} are each linked at least to a representative sensor. Thus, their data readings can be easily recovered through the spatial pre-interpolation method of stage 2. Whereas, the data readings of the sensors {2,3,4,7,15} are recovered thanks to the minimization (11) of stage 3.

## 8. Numerical Results

In this section, we first evaluate our proposed structured approach with the variation of the tuning parameter $fac_{1}$ of the minimization (11) of stage 3, while fixing $fac_{2}$, in order to measure the data reconstruction error ratio with respect to the different simulated values of $fac_{1}$ and choose the appropriate one that gives the lowest data recovery error. Secondly, we compare the performance of our proposed structured approach, with the fixed tuning parameter $fac_{1}$, to that of a benchmark scheme, which was designed basically on what was proposed in [6] and in line with our scenarios’ requirements. Indeed, at the end of their work, Xie et al. considered, in [6], that there is a small number of empty rows in *M*, which is, for N=196, 14 data rows were missing, namely 7% of *N* (i.e., 93% of *N* of representative sensors). As we have already stated at the beginning of this paper, treating an important number of missing rows has not been the main focus of their work. Thus, their proposed approach has not taken the existence of the isolated nodes in the network into account. In fact, they basically focused on the existence of successive missing or corrupted entries in the received data matrix *M*. However, to the best of our knowledge, this is the unique paper that has treated a similar case using MC, and with which we can compare our approach in the first part of this section. Subsequently, in the second part, we try to separately evaluate the benefits of each building block of the proposed approach, namely:

- Involving all of the detected clusters equitably in the sampling process.
- Selecting the representative sensor nodes using Algorithm 3.
- Adding the minimization (11) to the reconstruction pattern.

Making use of the generated signal of the example of Section 4, we perform our structured approach over different scenarios to illustrate the impact of these aforementioned techniques on the interpolation accuracy of the data matrix. To measure the reconstruction error, we opted for the following metrics, where *X* and $\hat{X}$ represent, respectively, the initial raw data matrix and the reconstructed one:

1—$NMAE_{tot}$: the Normalized Mean Absolute Error on all missing entries:

(13) $$NMAE_{tot}=\frac{\sum_{i,t:\Omega_{M}\left(i,t\right)=0}\left|X\left(i,t\right)-\hat{X}\left(i,t\right)\right|}{\sum_{i,t:\Omega_{M}\left(i,t\right)=0}\left|X\left(i,t\right)\right|}.$$

2—$NMAE_{MC}$: the Normalized Mean Absolute Error on the partially missing entries, which correspond to the non-transmitted readings of the representative nodes:

(14) $$NMAE_{MC}=\frac{\sum_{i,t:\left(i,t\right)\in \Omega_{mc}}\left|X\left(i,t\right)-\hat{X}\left(i,t\right)\right|}{\sum_{i,t:\left(i,t\right)\in \Omega_{mc}}\left|X\left(i,t\right)\right|},$$

where $\Omega_{mc}$ is the set of indexes of the partially missing entries, as found in the received data matrix $M\in {I\;R}^{N\times T}$.

3—$NMAE_{ER}$: the Normalized Mean Absolute Error on the missing entries of the fully empty rows, which correspond to the inactive sensor nodes’ readings:

(15) $$NMAE_{ER}=\frac{\sum_{i,t:i\in \Omega_{ER}}\left|X\left(i,t\right)-\hat{X}\left(i,t\right)\right|}{\sum_{i,t:i\in \Omega_{ER}}\left|X\left(i,t\right)\right|},$$

where $\Omega_{ER}$ is the set of indexes of the $\left(N-N_{rep}\right)$ empty rows, found in the received data matrix $M\in {I\;R}^{N\times T}$.

4—CR: the Compression Ratio:

(16) $$CR=\frac{N\times T-card(\Omega )}{N\times T},$$

where $\Omega =\left\{\left(i,t\right)\;|\;\Omega_{M}\left(i,t\right)=1\right\}$. Hence, card(Ω) denotes the number of observed entries in *M*.

We vary $pct_{Nrep}$ from 10 to 80, and, for each given $pct_{Nrep}$, we vary $pct_{m}$ from 10 to 80, in order to assess the proposed approach under different CRs. It is obvious that the range of the values of CR depends on the value assigned to $pct_{Nrep}$. The larger $pct_{Nrep}$, the higher CR range can be used. Note that we are mainly interested in the small values of $pct_{Nrep}$ and $pct_{m}$, since we are considering the high loss scenarios. Specifically, we consider that N=50 sensor nodes are randomly distributed in a square observation area of size 100 m×100 m, and we monitor the WSN during T=100 time slots.

**Figure 4:**
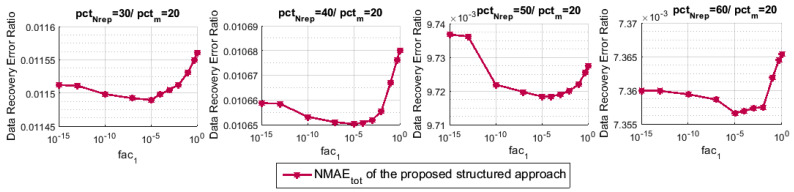
$NMAE_{tot}$ for the proposed technique with respect to the regularization parameter $fac_{1}$ of the minimization (11).

To begin, we measure the data reconstruction error ratio $NMAE_{tot}$ of our proposed structured approach with the variation of the regularization parameter $fac_{1}$. To do so, we fix $fac_{2}$ to 1, then, we accordingly adjust $fac_{1}$, which vary from the value 1 to the value $10^{-15}$. Note that we have used K=5 during all of the simulations of this paper. Figure 4 shows the effect of $fac_{1}$ on the data recovery performance of our approach. For $pct_{m}=20$, we vary $pct_{Nrep}$ and for each case the $NMAE_{tot}$ is calculated with respect to $fac_{1}$. As we can note, the minimization (11) of stage 3 typically performs better for the value $fac_{1}=10^{-5}$ than the other values. For that reason, we retain this value and use it in all of the next experiments.

**Figure 5:**
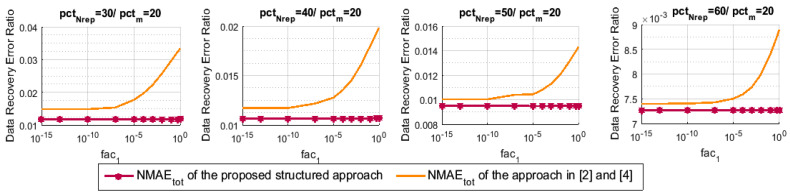
$NMAE_{tot}$ for the proposed technique and the one in [2,4].

In Section 7, the proposed minimization-based interpolation technique (11) of stage 3 has been investigated and then updated when compared to the one of our previous works ([2] Equation (8)) and ([4] Equation (5)), as we have mentioned. Figure 5 illustrates a performance comparison in terms of $NMAE_{tot}$ between the two methods for different values of $pct_{Nrep}$ and with respect to the regularization parameter $fac_{1}$. As we can clearly notice through the simulations of Figure 5, for different values of $fac_{1}$, the data recovery performance is highly improved with the proposed minimization-based interpolation technique of this paper compared to the one shown in our original papers.

**Figure 6:**
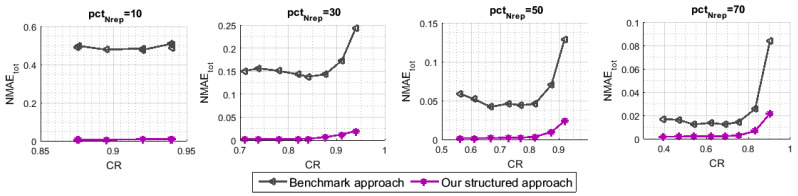
$NMAE_{tot}$ for the proposed technique and for the Benchmark.

In the third simulation, we implement a benchmark approach that is based on what was proposed in [6]. The sampling pattern of this approach consists in choosing the set $N_{rep}$ of representative sensor nodes in a purely random way, which is exactly the same as randomly selecting the empty rows. Likewise, for each time instant *t*, *m* nodes are uniformly selected from the set $N_{rep}$ to deliver their readings to the sink. Here, neither the selection of the representative sensors nor the selection of the transmitting ones takes the detected clusters into account. As for the reconstruction pattern, to obtain the final recovered data matrix $\hat{X}$, this approach performs the MC, and then the spatial pre-interpolation. The temporal pre-interpolation was omitted, since we do not consider the existence of empty columns in the observed data matrix *M* (This is not the case with our scenario, since, at every *t*, we ensure the transmission of *m* readings sensed in different *m* locations). In Figure 6, we have measured the $NMAE_{tot}$ with respect to the variation of CR, namely $pct_{m}$, for different values of $pct_{Nrep}$. Our approach distinctly outperforms the benchmark one across the entire ranges of CR, as we can note from the plots. We are able to go up to 90% of missing rows ($pct_{Nrep}=10$) with an interesting reconstruction performance, $NMAE_{tot}$ of about 0.008, while the benchmark technique yields an $NMAE_{tot}$ of [0.47,0.5].

**Figure 7:**
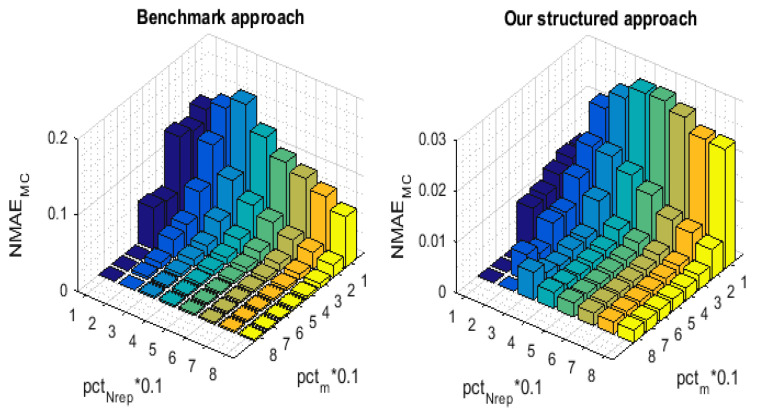
$NMAE_{MC}$ for the proposed technique and for the Benchmark.

**Figure 8:**
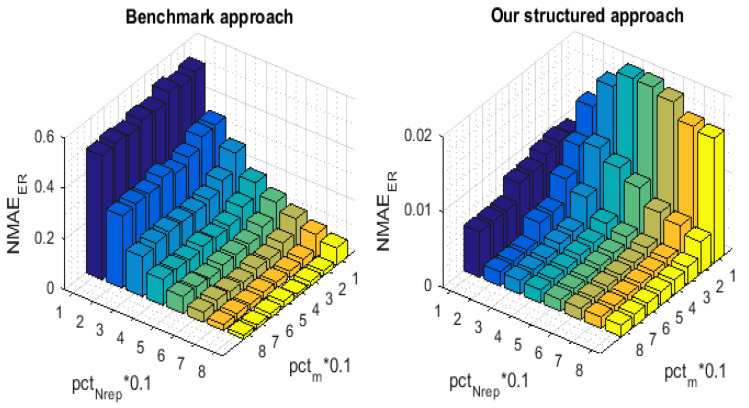
$NMAE_{ER}$ for the proposed technique and for the Benchmark.

Figure 7 and Figure 8 illustrate the 3-D bar graph of, respectively, the $NMAE_{MC}$ and the $NMAE_{ER}$ values with the variation of $pct_{Nrep}$ and $pct_{m}$. For the convenience of comparison, we have implemented the $NMAE_{MC}$ and $NMAE_{ER}$ in order to separate the error ratios and demonstrate the recovery performance enhancement that has been achieved by our proposed approach on, respectively, the partially and fully missing readings.

**Figure 9:**
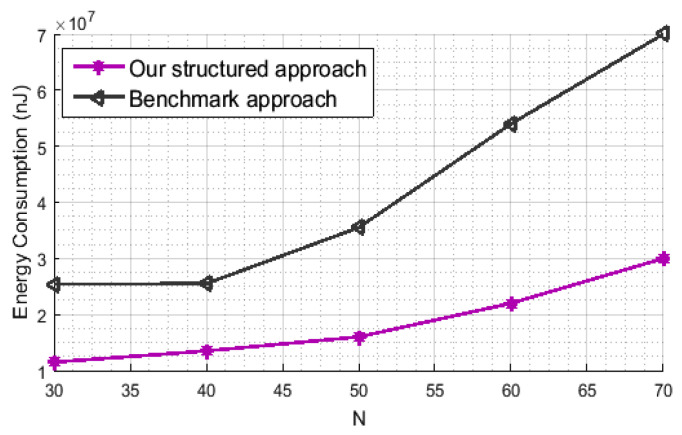
Energy consumption for the proposed technique and for the Benchmark.

Note that the considered framework extremely reduces the overall network energy consumption, since we only use a small set of representative sensors for the data transmission. Furthermore, when compared to the benchmark approach, the proposed one can further improve the sensors lifetime. In fact, for a given $NMAE_{tot}$ target of 0.02 and $pct_{Nrep}=60$, we compute the energy consumption during the *T* time instants for the both compared approaches, depending on the number *N* of sensors. In this simulation, we consider that two nodes *i* and *j* can directly communicate with each other, without the need for relaying, only if the Euclidean distance $dst_{i,j}$ between them is within some transmission radius (r) that scales with $\Theta (\sqrt{logN/N})$ [21]. To route the data towards the sink node, we perform the shortest path tree that was computed by the Dijkstra algorithm [16]. The following model is used in order to compute the energy consumption during data transmission [45].

(17) $$\left\{\begin{array}{c} E_{Tx}\left(L,dst_{i,j}\right)=E_{elec}\times L+\varepsilon_{amp}\times L\times dst_{i,j}^{2}\\ E_{Rx}\left(L\right)=E_{elec}\times L, \end{array}\right.$$

where $E_{Tx}\left(L,dst_{i,j}\right)$ and $E_{Rx}\left(L\right)$ represent, respectively, the amount of energy that is consumed by a specific node *i*, to deliver or receive an *L*-bit packet through a distance of length $dst_{i,j}$. In (17), $E_{elec}$ is the energy that is required by the transceiver circuitry at the sender or the receiver and $\varepsilon_{amp}$ is the energy consumed by the transmitter’s amplifier. Regarding the parameters setting, L=120 bits [15], $E_{elec}=$ 50 nJ/bit and $\varepsilon_{amp}=$ 100 pJ/bit/m${}^{2}$ [45]. Figure 9 illustrates the energy consumption for the proposed framework as well as for the benchmark one. Indeed, our approach requires far less sensor nodes’ readings, consequently, much less energy consumption, in order to achieve the same reconstruction performance.

**Figure 10:**
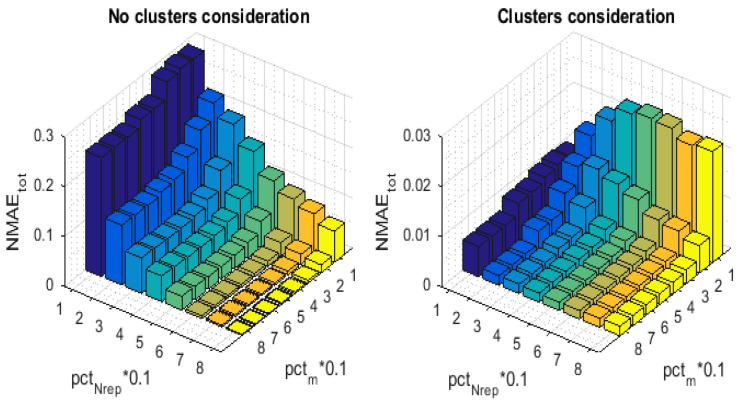
$NMAE_{tot}$ with and without clusters consideration.

Let us now focus on the benefits of the clusters selection. We show that taking the detected clusters during the representative nodes selection process as well as during the assignment of the sensing and transmitting schedule into account significantly ameliorates the data recovery performance. Thus, we compare our approach to another one, for which we proceed, regardless the existence of the different clusters. The set $N_{rep}$ of representative sensor nodes is selected according to (7) instead of (9), i.e., the spatial correlation criteria are present during the node selection process. Nevertheless, we do not have equitable representation of the different regions that compose the whole network. Withal, for each *t*, the *m* transmitting nodes are picked from the set $N_{rep}$ in a purely random way to sense then deliver their data readings, i.e., $m=pct_{m}\%\times N_{rep}$ instead of (10). To recover the received data matrix, both algorithms apply the 3-stage reconstruction pattern of Section 7. Figure 10 illustrates the 3-D bar graph of the $NMAE_{tot}$ values with the variation of $pct_{Nrep}$ and $pct_{m}$. This simulation shows how curiously interesting the clusters consideration is. The barres depict that our approach provides a considerable improvement in terms of $NMAE_{tot}$ when compared to the algorithm of comparison, especially in the high compression ratios, i.e., when the number of transmitting sensor nodes is very limited. Note that without enforcing the involvement of all the clusters in the data sensing and transmission process, sensor nodes that belong to the small clusters could be totally ignored, which gravely deteriorates the recovery process.

**Figure 11:**
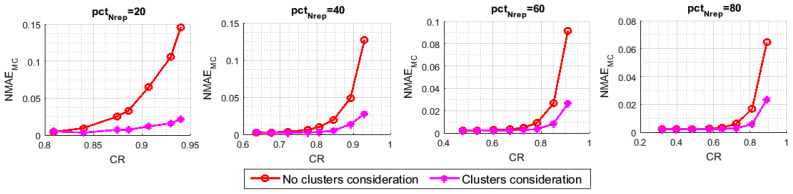
$NMAE_{MC}$ with and without clusters consideration.

**Figure 12:**
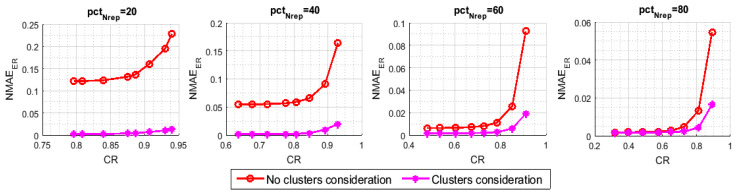
$NMAE_{ER}$ with and without clusters consideration.

In Figure 11 and Figure 12, we have measured, respectively, the $NMAE_{MC}$ and the $NMAE_{ER}$ with respect to the variation of CR, namely $pct_{m}$, for different values of $pct_{Nrep}$. Figure 11 and Figure 12 highlight the effect of the introduced block on the recovery of, respectively, the representative nodes’ and the inactive nodes’ readings. Although both of the techniques apply the same MC resolution method, the $NMAE_{MC}$ of our approach is much lower than that of the benchmark. The $NMAE_{ER}$ also seems to be heavily affected, despite the fact that the clusters consideration, at the base, only targets the first stage of the reconstruction pattern, which is the MC resolution. For example, with $\left(pct_{Nrep}=20,pct_{m}=10\right)$, $\left(pct_{Nrep}=40,pct_{m}=10\right)$, and $\left(pct_{Nrep}=60,pct_{m}=10\right)$, we can reach an improvement respectively of 93.88%, 87.87%, and 79.38%, when we enforce the involvement of all the clusters in the data sensing and transmission.

**Figure 13:**
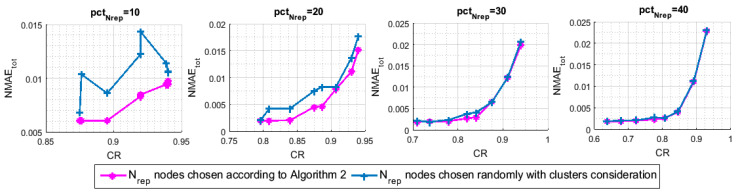
The impact of the representative node selection technique on the $NMAE_{tot}$.

**Figure 14:**
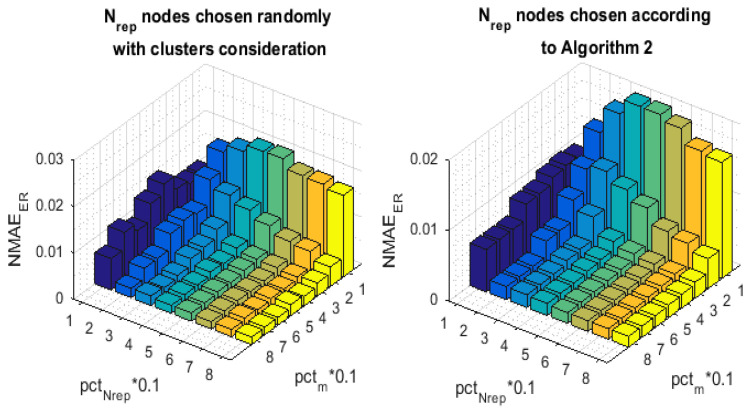
The impact of the representative node selection technique on the $NMAE_{ER}$.

The next scenario aims to prove the importance of neatly selecting the $N_{rep}$ representative nodes. Making use of the spatial correlation in the selection process, as detailed in Algorithm 3, these nodes are selected under the criterion of having the best representation of the whole network. We compare our algorithm to another one that selects its representative nodes randomly in order to investigate the efficiency of the proposed selection process. However, in order to be comparable, this one takes the existing clusters when selecting its representative nodes into account. Hence, the set $N_{rep}$ of representative nodes consists of the combination of *J* subsets, ${\left(N_{rep_{j}}\right)}_{j=1,\ldots ,J}$, where $N_{rep_{j}}$ includes $N_{rep_{j}}$ representative nodes selected randomly from cluster $CL_{j}$ while using the same shared percentage $pct_{Nrep}$, where $N_{rep}=\sum_{j=1}^{J}N_{rep_{j}}$ and $N_{rep_{j}}=pct_{Nrep}\%\times cl_{j}$. Both of algorithms design their sensing and transmitting schedules, $\Omega_{M}\in {I\;R}^{N\times T}$, based on their selected sets $N_{rep}$ of representative nodes, as described in Section 6 and according to (10). To recover the received data matrix, both of the performed algorithms apply the three-stage reconstruction pattern of Section 7. Figure 13 and Figure 14 depict the results of this simulation. Figure 13 illustrates the $NMAE_{tot}$. As we can see, when compared to the random selection process, the selection scheme of Algorithm 3 provides a considerable improvement in terms of $NMAE_{tot}$ for the high CRs. The gap between the two curves decreases as we increase the number $N_{rep}$ of representative nodes, namely $pct_{Nrep}$, since we decrease the probability of choosing different sets $N_{rep}$.

Let us focus on Figure 14, which highlights the $NMAE_{ER}$ to reveal the impact of our selection process on the reconstruction performance of the empty rows. Expectedly, we find that the $NMAE_{ER}$ is sensitive to the used selection method, which confirms the aforementioned hypothesis. That is, in order to guarantee an accurate reconstruction for the inactive nodes missing data, great care must be taken when selecting the set $N_{rep}$.

**Figure 15:**
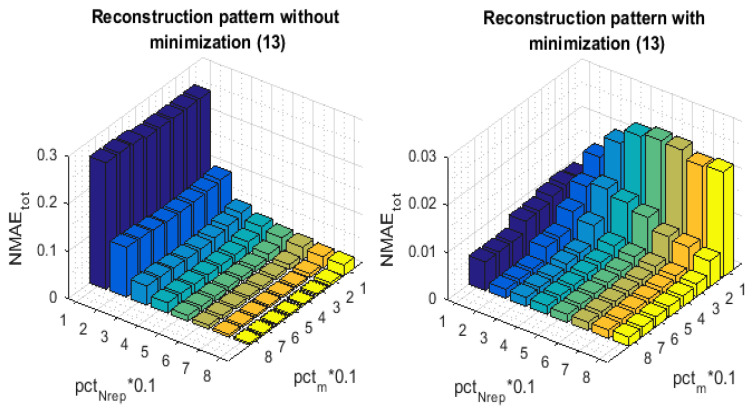
The impact of spatial interpolation technique on the $NMAE_{tot}$.

**Figure 16:**
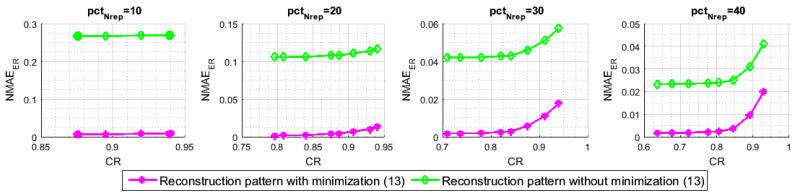
The impact of spatial interpolation technique on the $NMAE_{ER}$.

The last simulation highlights the benefit of the 3^rd^ stage of the proposed reconstruction pattern. We compare our algorithm to the one that only uses the first two stages of Section 7 to obtain its final recovered data matrix $\hat{X}$. Following the same logic of the previous experiences, in order to be comparable, we use the sampling pattern of Section 6 with both of the simulated algorithms, which yields the same set $N_{rep}$ of representative nodes and, consequently, the same set of inactive nodes. Noticeably, we can detect a considerable gap in terms of $NMAE_{tot}$ between the barres of Figure 15. This difference for all of the $pct_{Nrep}$ values comes from the non-reconstructed readings of the $N_{Is}$isolated nodes with the algorithm of comparison. Because we simulated the same network with the same sensor nodes neighboring, the set of the $N_{Is}$isolated nodes is the same for both of the compared algorithms. Figure 16, which depicts the $NMAE_{ER}$ for both approaches, illustrates that we can reduce the reconstruction error of the empty rows up to 96.89% for $\left(pct_{Nrep}=10,pct_{m}=40\right)$, 96.08% for $\left(pct_{Nrep}=20,pct_{m}=40\right)$, 93.65% for $\left(pct_{Nrep}=30,pct_{m}=40\right)$ and 90.2% for $\left(pct_{Nrep}=40,pct_{m}=40\right)$, when we apply the minimization (11). These results show that the number of isolated nodes is important for a small $pct_{Nrep}$. Hence, adding a third interpolation technique, as our proposed minimization (11), becomes heavily needed. Otherwise, we end with a data matrix, which is almost half built, even less.

## 9. Conclusions

In this paper, we have investigated an interesting challenge in the dense WSNs. In fact, we have proposed letting a significant number of sensor nodes remain idle. Subsequently, relying on a novel MC-based reconstruction framework, we recover their readings based on the received ones. The strength of our approach lies in its integration or inclusivity for both the compression and reconstruction patterns. For the sampling part, by making use of the inter-spatial correlation feature, we have presented a strategy that neatly selects a restricted number of representative sensor nodes under the criterion of having the best representation of the whole network. Subsequently, for each cluster, we schedule where and when to sense the field. As for the reconstruction part, by taking advantage of the readings similarities in WSNs, we propose an optimization technique that is annexed to the MC resolution. This method, which is positioned in the third stage of the recovery operation, guarantees the reconstruction of all the empty rows corresponding to the omitted sensor nodes. Altogether, these techniques succeed in handling the aforementioned high loss scenario. We have obtained satisfactory results proving the efficiency and the robustness of the proposed techniques as well as the whole unified approach. The results, which were obtained with the multi-Gaussian generated signal, outperform all of the state of the art techniques. They revealed that we are able to go up to 90% of missing rows (i.e. only 10% of *N* of representative sensor nodes), while we still achieve an interesting reconstruction performance by giving a $NMAE_{tot}$ of about 0.008 when compared to the benchmark one, which is still within the range of [0.47,0.5].
